# CO and HCHO Sensing by Single Au Atom-Decorated WS_2_ Monolayer for Diagnosis of Thermal Aging Faults in the Dry-Type Reactor: A First-Principles Study

**DOI:** 10.3390/ma17051173

**Published:** 2024-03-02

**Authors:** Qi Zhao, Yuyan Man, Jin He, Songyuan Li, Lin Li

**Affiliations:** 1 State Grid Tianjin Electric Power Research Institute, Tianjin 300384, China; 2 Tianjin Key Laboratory of Internet of Things in Electricity, Tianjin 300384, China; 3 State Grid Tianjin Electric Power Company, Tianjin 300232, China

**Keywords:** gas-sensing mechanism, Au-WS_2_ monolayer, dry-type reactors, thermal aging

## Abstract

CO and HCHO are the main pyrolysis gases in long-term running dry-type reactors, and thus the diagnosis of thermal insulation faults inside such devices can be realized by sensing these gases. In this paper, a single Au atom-decorated WS_2_ (Au-WS_2_) monolayer is proposed as an original sensing material for CO or HCHO detection to evaluate the operation status of dry-type reactors. It was found that the Au atom prefers to be adsorbed at the top of the S atom of the pristine WS_2_ monolayer, wherein the binding force is calculated as −3.12 eV. The Au-WS_2_ monolayer behaves by chemisorption upon the introduction of CO and HCHO molecules, with the adsorption energies of −0.82 and −1.01 eV, respectively. The charge density difference was used to analyze the charge-transfer and bonding behaviors in the gas adsorptions, and the analysis of density of state as well as band structure indicate gas-sensing mechanisms. As calculated, the sensing responses of the Au-WS_2_ monolayer upon CO and HCHO molecule introduction were 58.7% and −74.4%, with recovery times of 0.01 s and 11.86 s, respectively. These findings reveal the favorable potential of the Au-WS_2_ monolayer to be a reusable and room-temperature sensing candidate for CO and HCHO detections. Moreover, the work function of the Au-WS_2_ monolayer was decreased by 13.0% after the adsorption of CO molecules, while it increased by 1.2% after the adsorption of HCHO molecules, which implies its possibility to be a work-function-based gas sensor for CO detection. This theoretical report paves the way for further investigations into WS_2_-based gas sensors in some other fields, and it is our hope that our findings can stimulate more reports on novel gas-sensing materials for application in evaluating the operation conditions of dry-type reactors.

## 1. Introduction

Dry-type reactors contribute largely in power systems for the purposes of current limiting in series circuits and reactive compensation in parallel circuits [1,2]. Differently from oil-immersed transformers, wherein dielectric oil is adopted as the insulation medium [3], in dry-type reactors the epoxy resin is employed as the insulation medium (solid material) to prevent the possible insulating defects within devices with favorable insulation performance [4]. When insulation faults (partial overheat and partial discharge) occur, the epoxy resin undertakes the power energy supply, thus guaranteeing the good running of dry-type reactors. Nevertheless, the abundant power energy produced by these insulation faults can decompose the epoxy resin in a long running dry-type device, forming certain noxious gases, among which CO and HCHO account for the major amount [5,6]. Due to the decomposition of epoxy resin, their insulation performance would be impaired, similar to the weakened insulation performance of the transformer oil, which would generate some typical gases under certain insulation faults [7,8,9]; and due to the generation of these toxic gases, the electrical substation would be pervaded with them everywhere, therefore threatening the health of the maintenance workers [10]. From this aspect, researchers in the field of gas sensing put forward the exploration of CO or HCHO gas sensors in order to realize the purposes of (i) evaluating the decomposing conditions of epoxy resin and (ii) ensuring the safety of working environments for maintenance people.

For the exploration of typical gas sensors, nano-devices prepared from 2D materials have been frequently investigated recently, and have been proven to have favorable sensitivity and quick response capabilities [11,12,13], which could be a result of their unique properties of large surface area, admirable electron mobility, and favorable chemical reactivity towards gaseous molecules [14,15,16]. Typically, transition metal dichalcogenides (TMDs) and their 2D structures have been widely reported on in terms of gas adsorptions and detections, based on theoretical calculations [17,18,19,20]. As proven, these newly discovered 2D materials with semiconducting property undertake admirable chemical interactions with gas molecules and show comparable sensing performance to graphene [21]. This provides them with admirable potential as novel semiconducting devices with tunable electronic properties for gas-sensing applications. Specifically, the WS_2_ monolayer, as a 2D layered TMD, has been explored for application in the fields of optical and photoelectric sensors [21,22]. In addition, the WS_2_ monolayer has been successfully synthesized by many novel methods, such as chemical vapor deposition at low pressure and sulfurization with WO_3_ thin film [23,24]. On the other hand, the gas-sensing performance of the WS_2_ monolayer has been less widely explored. Importantly, considering the favorable and tunable electronic properties of the WS_2_ monolayer, we presume that through the surface decoration technique, especially by use of a noble metal atom, the decorated WS_2_ monolayer could show adsorption and sensing behaviors toward the gas molecules. In this regard, the application of WS_2_-based materials could be largely broadened, which would be beneficial to exploring novel gas-sensing materials as well. Therefore, we would like to perform first principles calculations, using theoretical simulations, to give a first insight into such an assumption. We would also like to state that the theoretical simulations performed upon the gas-sensing material are also significant in helping us understand the adsorption behavior and sensing mechanism.

In this work, we propose a single Au atom-decorated WS_2_ (Au-WS_2_) monolayer as a novel gas-sensing material for the detection of CO and HCHO molecules. We should mention that the Au-WS_2_ configuration is established by the adsorption of a single Au atom onto a pristine WS_2_ monolayer, and the gas adsorption and sensing mechanisms are expounded based on the first principles theory. The Au metal was employed in this work to decorate the WS_2_ surface as a result of its favorable and effective catalytic properties in gas–surface reactions [25]. Thereby, we presume that Au decoration could enable admirable sensing responses towards two such gas species. Also, we are confident that the findings in this work can offer some guidance towards deeper investigations into the WS_2_ monolayer and its related materials as gas sensors; furthermore, materials with high sensitivity towards typical gases can be applied for the fault monitoring of the electrical devices in the power systems.

## 2. Computation Methods

The first-principles simulations in this report were all implemented within the DMol^3^ package (Materials Studio 8.0) [26]. The Perdew–Burke–Ernzerhof (PBE) exchange function was employed to consider the correlation terms and electron exchange [27]. We adopted the Tkatchenko and Scheffler DFT-D2 method to deal with the Van der Waals forces and long-range interactions [28]. The *k*-point mesh of the Monkhorst-Pack was set as 10 × 10 × 1 for geometric and electronic calculations. Moreover, the energy tolerance accuracy (namely, geometric convergence) was set as 10^−5^ Ha, and the self-consistent loop energy as well as global orbital cut-off radius were defined as 10^−6^ Ha and 5.0 Å, respectively [29]. All the calculations are spin-polarized.

Upon the Au-decoration and the gas adsorption processes, we established a 3 × 3 × 1 supercell for the pristine WS_2_ monolayer as the nano-substrate with 9 W atoms and 18 S atoms. We determined a vacuum region of 15 Å in order to prevent possible interaction between the adjacent units around the supercell [30]. As one Au atom was adsorbed onto the WS_2_ monolayer, we could calculate that the Au-decorating concentration was 3.7%. Also, the charge-transfer property in these two processes was analyzed by using the Hirshfeld population method. Based on this definition, we would consider the atomic charge of the Au adatom (*Q*_Au_) and the molecular charge of the adsorbed molecule (*Q*_T_) in each configuration. It should be mentioned that a positive value of *Q*_Au_ or *Q*_T_ indicates an electron-donating behavior, whereas a negative value suggests the electron-accepting property.

## 3. Results and Discussion

### 3.1. Au Decoration Effects on the WS_2_ Monolayer

**Figure 1:**
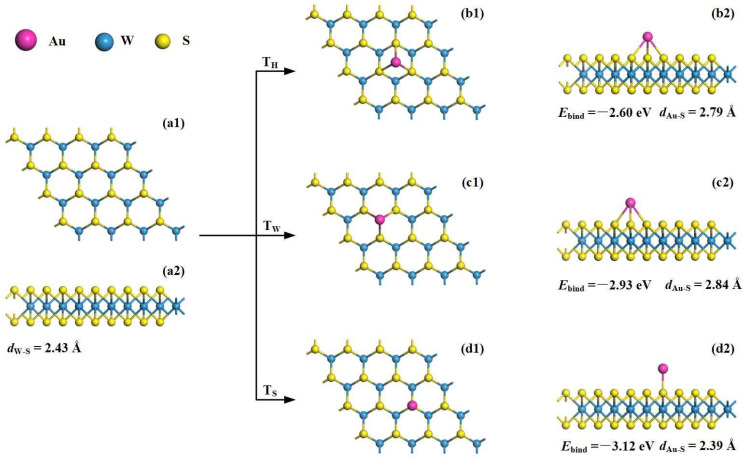
Au-decoration process on the WS_2_ monolayer: (**a1**,**a2**) morphology of pristine WS_2_; (**b1**,**b2**) Au decorating above the center of the hexagonal ring of WS_2_; (**c1**,**c2**) Au decorating at the top of one W atom and (**d1**,**d2**) Au decorating at the top of one S atom.

Figure 1 presents the Au decoration process on the pristine WS_2_ monolayer. To complete this process, we firstly performed geometric optimization for the configuration of the pristine WS_2_ monolayer. Then, the effects of Au decoration on the optimized substrate were investigated. In terms of Au decoration, three possible sites could be considered: T_H_ (above the center of the hexagonal ring of WS_2_), T_w_ (at the top of the W atom) and T_s_ (at the top of the S atom), respectively. To evaluate the binding force between the Au adatom and the WS_2_ surface, the binding energy (*E*_bind_) between them is formulated, expressed as [31]:

(1) $$E_{bind}=E_{Au-{WS}_{2}}-E_{Au}-E_{{WS}_{2}}$$

where the *E*_Au-WS2_, *E*_Au_ and *E*_WS2_ are the total energy of the Au-WS_2_ system, isolated Au atom and pristine WS_2_ monolayer. In the pristine WS_2_ monolayer, the W–S bond length is measured as 2.43 Å, and the lattice constant is obtained as 3.18 Å, which are in good agreement with the previous results for the monolayer WS_2_ of 2.43 and 3.19 Å [32,33]. These findings imply the calculation accuracy of this work.

To determine the most stable decoration position, the *E*_bind_ was calculated for all three of the sites considered above. After calculation, it was found that the absolute *E*_bind_ was the highest at the T_S_ site. The *E*_bind_ has an absolute value of 3.12 eV, which is slightly larger than that of 2.93 eV for the T_W_ site, and much larger than that of 2.60 eV for the T_H_ site. Meanwhile, the shortest Au–S bond at the T_S_ site (2.39 Å) in comparison with those at the T_W_ site (2.84 Å) and T_H_ site (2.79 Å) also reveals the strongest Au–S bonding force at the T_S_ site. Therefore, we determine that Au decoration at the T_S_ site is the most preferred configuration for the Au-WS_2_ monolayer. It should be noted that there are Au–S bonds in all three configurations, indicating the good binding force between the Au adatom and WS_2_ monolayer. For the determined Au-WS_2_ configuration, it can be seen that the Au atom formed one covalent bond with the S atom on the upper layer of the WS_2_ with the bond length of 2.58 Å. Compared with the pure WS_2_ monolayer, the W–S bonds nearing the decorating site suffered small deformations in the Au-WS_2_ structure, and the W–S bonds in the Au adatom were measured to be 2.43 Å, which indicates that no significant remodeling was observed on the WS_2_ monolayer. Accordingly, we should adopt the configuration of Au decoration on the T_S_ site of the WS_2_ monolayer as the so-called Au-WS_2_ monolayer for the latter simulations of electronic properties and adsorption processes.

**Figure 2:**
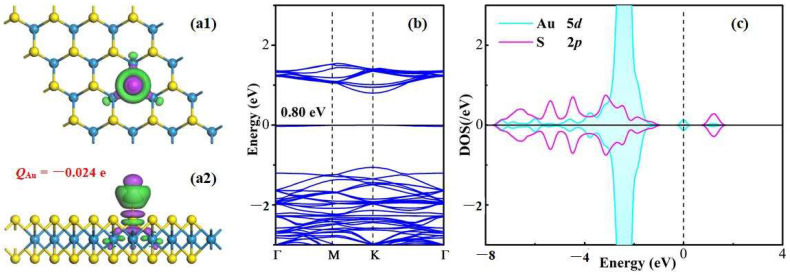
(**a1**,**a2**) CDD, (**b**) BS and (**c**) atomic DOS of Au-WS_2_ system. In CDD, the green areas signify electron accumulation and the purple areas signify electron depletion, with the iso-surface of 0.008 e/Å^3^. In BS, the black value indicates the bandgap and in DOS the dashed line signifies the Fermi level.

The charge density difference (CDD), band structure (BS) as well as atomic density of state (DOS) of the Au-WS_2_ system are exhibited in Figure 2. It should be noted that the spin up and spin down of the Au-WS_2_ monolayer are symmetric in the BS distributions. Therefore, only the spin up of this system is plotted in the figure. Based on the CDD, it can be found that there was dense electron accumulation in the Au–S bond, which suggests the favorable electron hybridization between the S atom and the Au adatom. According to Hirshfeld analysis, the Au adatom is charged by −0.024 e, revealing that 0.024 e transfers from the WS_2_ surface to the Au adatom. The charge transfer enhances the formation of the Au–S bond, where electron localization occurs. According to the BS of the Au-WS_2_ system, it could be observed that the bandgap was 0.80 eV. One should note that the bandgap of the pristine WS_2_ monolayer was obtained as 1.85 eV using the PBE method here, which is quite close to the previous findings, ranging from 1.80 eV to 1.85 eV [22,23], revealing the good accuracy of the current work. Therefore, this work will further use the PBE function in the following calculations. Besides this, the top valence band and the bottom conduction band of the Au-WS_2_ monolayer are both located at the K point. These results manifest a direct semiconducting property in the Au-WS_2_ monolayer. Moreover, in the atomic DOS of the Au-WS_2_ system, the Au 5*d* orbital is strongly hybridized with the S 2*p* orbital at −7.2~−1.0 and 1.4 eV, which indicates the desirable orbital interactions between the Au adatom and the S atom, thus expounding the strong binding force in the new-formed Au–S bond.

### 3.2. Adsorption Behavior of Au-WS_2_ Monolayer

The adsorption of CO and HCHO molecules was induced to study the adsorption behavior of the Au-WS_2_ monolayer, especially highlighting the role of the Au adatom. For CO adsorption, the configurations include C-end orientation, O-end orientation and CO molecular parallel orientation upon the Au atom. For HCHO adsorption, the configurations include C-O bond orientation, C=C bond orientation and O-end orientation. After full optimizations, the most stable configuration (MSC) for gas adsorption on the Au-WS_2_ monolayer is identified as the one with the most negative value of adsorption energy (*E*_ad_), calculated by Equation (2):

(2) $$E_{ad}=E_{Au-WS_{2}/gas}-E_{Au-WS_{2}}-E_{gas}$$

**Figure 3:**
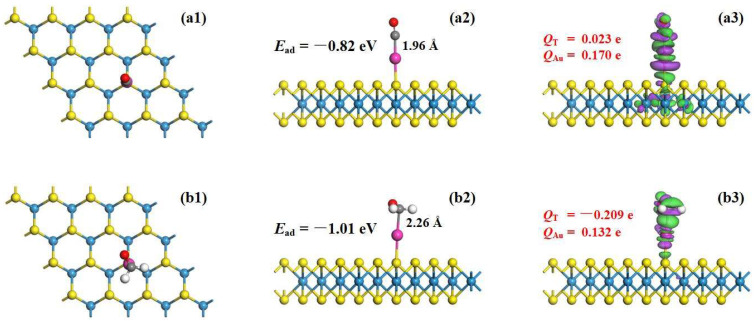
(**a1**,**a2**,**b1**,**b2**) CO and HCHO adsorbed systems. MSC and (**a3**,**b3**) CDD. The set of CDD is the same as Figure 2.

$E_{Au-WS_{2}/gas}$ and *E*_gas_ indicate energies of the Au-WS_2_/gas system and the gas molecule, respectively. After geometric optimizations and energy calculations, the MSC for CO and HCHO adsorptions on the Au-WS_2_ monolayer can be obtained. The MSC are adopted for further analysis of the geometric and electronic properties of the Au-WS_2_ monolayer for adsorptions of CO and HCHO molecules. Therefore, the MSC and related CDD are plotted in Figure 3 to illustrate the adsorption performance and electron redistribution in the CO and HCHO systems.

For CO adsorption on the Au-WS_2_ surface, it can be seen that the adsorbed CO molecule stands above the Au adatom via the C-end position. This means that the C atom of the CO molecule is trapped by the Au adatom with the Au–C bond formed measured as 1.96 Å. The CO molecule stands vertically above the WS_2_ monolayer. Such a configuration is quite different from the Rh-PtSe_2_/CO configuration, in which the adsorbed CO molecule significantly slopes away from the PtSe_2_ monolayer [4]. One should notice that the length of the new-formed Au–C bond (1.96 Å) is slightly short than that of the summed covalent radii of C and Au atoms (2.11 Å [34]), which reveals the strong Au–C binding force. Also, the Au–S bond in the Au-WS_2_/CO system is measured to be 2.33 Å, slightly shorter than that of 2.39 Å in the isolated Au-WS_2_ system, which indicates the good geometric stability of the Au-WS_2_ monolayer. Meanwhile, the *E*_ad_ of −0.82 eV also reflects the good adsorption behavior of the Au-WS_2_ monolayer upon CO. Combined with the shorter bond length and the larger absolute *E*_ad_ value compared with the critical value of 0.80 eV [35], the Au-WS_2_/CO system has been found to undergo chemisorption. Based on the Hirshfeld analysis, the Au adatom is positively charged by 0.170 e, and the adsorbed CO molecule is positively charged by 0.023 e. These findings imply the electron-releasing property of the CO molecule, which donates 0.023 e to the Au-WS_2_ surface. The Au adatom, which releases 0.194 e, also behaves as an electron-donator after CO adsorption. The adsorbed CO molecule releases 0.023 e, and the WS_2_ surface accepts a total of 0.217 e. From the CDD, the adsorbed CO molecule as well as the Au adatom are embraced by electron depletion, whereas the Au–C and Au–S bonds are surrounded by electron accumulations. Such distributions verify the electron-donating property of the CO molecule and Au atom (consistent with the Hirshfeld analysis). This phenomenon accords with the favorable binding force of the Au–C bond and the enhanced binding force of the Au–S bond.

For HCHO adsorption on the Au-WS_2_ surface, it is seen that the C atom of the HCHO molecule is trapped by the Au adatom, which is similar to that in the CO adsorbed system. The C atom is trapped by the Au adatom, thus forming a Au−C bond (measured to be 2.26 Å). In addition, the HCHO molecular plane is basically parallel to the WS_2_ surface. The Au–S bond length is measured as 2.40 Å, which is slightly longer than that of 2.39 Å in the isolated Au-WS_2_ system. This indicates a slightly weakened binding force of Au–S for HCHO adsorption. The *E*_ad_ in the HCHO system is more negative that in the CO system, revealing the stronger adsorption effect of the Au-WS_2_ monolayer upon HCHO molecule. Therefore, chemisorption can be inferred for the effect of the Au-WS_2_ monolayer adsorbing the HCHO molecule. Based on the Hirshfeld analysis, the adsorbed HCHO molecule is negatively charged by 0.209 e, whereas the Au adatom is positively charged by 0.132e. This implies that the Au adatom and WS_2_ monolayer release 0.156 e and 0.053 e, accounting for the charged value of −0.209 e of the adsorbed HCHO molecule. For the CDD of the HCHO adsorbed system, electrons are apparently accumulated on the HCHO molecule, while the Au atom exhibits electron depletion. Electron accumulation is also shown on the new-formed Au–C bond, revealing its favorable electron hybridization. However, the electron depletion on the Au–S bond suggests its weakness after the HCHO adsorption.

Although the processes of CO and HCHO adsorption are both chemisorption, the Au-WS_2_ monolayer shows a stronger adsorption performance upon HCHO. When interacting with Au-WS_2_, the CO molecule behaves as an electron-donator while the HCHO molecule is an electron-acceptor. This adsorption performance deforms the electronic property of the Au-WS_2_ monolayer, which will be further illustrated in the next section.

### 3.3. Electronic Property of Au-WS_2_ Monolayer in Gas Adsorptions

**Figure 4:**
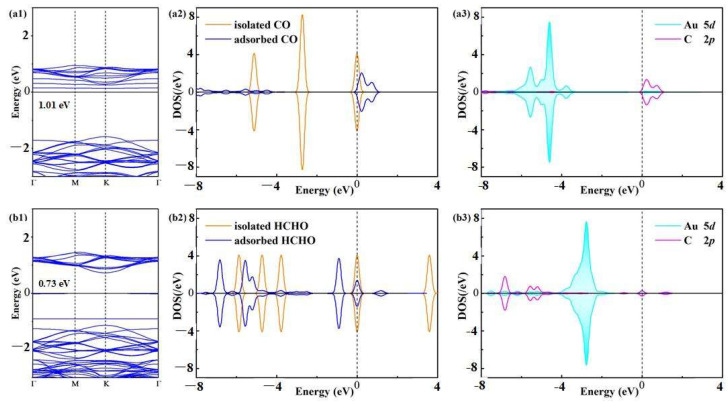
(**a1**–**a3**) BS, molecular DOS and atomic DOS of CO system and (**b1**–**b3**) HCHO system. The sets in BS and DOS are the same as in Figure 2 (The blue lines in (**a1**,**b1**) are the density of states of adsorbed gas species).

The BS, molecular DOS and atomic DOS are shown in Figure 4 to highlight the deformed electronic properties of the Au-WS_2_ monolayer in the CO and HCHO adsorption process. As the spinning up and spinning down of the gas adsorption systems are symmetric, the spinning up is also shown in this figure.

For the BS of CO and HCHO adsorption systems, shown in Figure 4a1,b1, the bandgaps are 1.01 and 0.73 eV, respectively. The top valence band and bottom conduction band are both located at the same point. These findings elucidate the direct semiconducting properties of the CO and HCHO adsorption systems, which means that the adsorption of the CO and HCHO molecules does not impact the direct semiconducting property of the Au-WS_2_ system. In addition, the bandgap of the Au-WS_2_ monolayer is increased by 0.21 eV (+25.9%) after the adsorption of the CO molecule, while it is decreased by 0.07 eV (−8.75%) after the adsorption of the HCHO molecule. This means that the electrical conductivity (*σ*) of the Au-WS_2_ monolayer would be decreased in the CO atmosphere and increased in the HCHO atmosphere, according to Equation (3) [36], in which *B_g_* is the bandgap of a certain material, λ is the constant, k is the Boltzmann constant and *T* is temperature:

(3) $$\sigma =\lambda \cdot e^{\left(-B_{g}/2kT\right)}$$

The variation in the electrical conductivity of the Au-WS_2_ monolayer provides the basic evidence required to explore it as a resistance-type gas sensor. The details regarding the exploration of CO and HCHO are explicated in the next section. It should be pointed out that the variation is caused by the adsorption of gas species. On the other hand, the electronic properties of the gas species are also impacted after interacting with the adsorbent surface.

Figure 4a2,b2 shows that the molecular electronic states of the isolated CO and HCHO are split into several small states after adsorptions. The split states are right-shifted in the CO molecule and left-shifted in the HCHO molecule. Besides this, there are several novel states around the Fermi level, which dominantly impact the electronic properties of the Au-WS_2_ system. Specifically, the states at 0.45 and 0.92 eV of the adsorbed molecule as well as those at −1.12, −0.18 and 0.98 eV contribute largely to the total DOS of the gas adsorbed systems [37].

In terms of the orbital DOS in Figure 4a3,b3, the Au 5*d* orbital is hybridized with the C 2*p* orbital of the adsorbed CO molecule at 0.45 and 0.92 eV. And the hybridization state for the Au 5*d* orbital and the C 2*p* orbital of the adsorbed HCHO molecule is located at −0.18 eV. As these states are also the novel states of the adsorbed gases discussed above, we can infer that the adsorbed molecules contribute largely to the whole DOS of the gas adsorbed system. Besides this, electron hybridization in the HCHO systems can also be found at −7.02, −5.76, −5.40 and 0.98 eV, which implies the stronger binding force in the newly formed Au–C bond in the Au-WS_2_/HCHO system in comparison with that in the Au-WS_2_/CO system. Moreover, the electron hybridizations between the Au 5*d* and C 2*p* orbitals verify the favorable orbital interaction as well as the strong binding force of the newly formed Au–C bonds in the CO and HCHO systems, which is in good accordance with the CDD distributions.

### 3.4. Gas Sensor Exploration for Au-WS_2_ Monolayer

In Section 3.3, it is shown that the bandgap of the Au-WS_2_ monolayer is increased by +25.9% after CO adsorption, and is decreased by −8.75% after HCHO adsorption. This finding reveals the possibility of exploring the Au-WS_2_ monolayer as a resistance-type gas sensor. For a resistance-type gas sensor, the sensing response (*S*) is calculated using Equation (4) [38]:

(4) $$S=\frac{\sigma_{gas}^{-1}-\sigma_{pure}^{-1}}{\sigma_{pure}^{-1}}$$

in which the *σ*_gas_ and *σ*_pure_ are the electrical conductivity of the gas adsorbed systems and the isolated nanomaterial, which can be calculated using Equation (3). Accordingly, the sensitivity of responses to the detection of CO and HCHO molecules when using the Au-WS_2_ monolayer are calculated to be 58.7% and −74.4%. Based on this theoretical analysis, the Au-WS_2_ monolayer displays a positive sensing response to the CO molecule and a negative one to the HCHO molecule. These sensing responses are both favorable to their detection using certain platform, e.g., an electrochemical workstation [39]. In addition, the Au-WS_2_ monolayer shows a stronger sensing performance towards HCHO compared with CO. So the opposite sensing tendency contributes to the selective detections of CO and HCHO. Therefore, the Au-WS_2_ monolayer shows potential for use as a sensing material to detect CO and HCHO molecules sensitively and selectively [40].

If it is to be used as a reusable gas sensor, the recovery property of the sensing material should be taken into consideration. As expressed in Equation (5), the recovery time (*τ*) could be obtained by using the adsorption energy (*E*_ad_) based on the van’t Hoff–Arrhenius theory [41]:

(5) $$\tau =A^{-1}e^{\left(-E_{ad}/kT\right)}$$

In this equation, A is the attempt frequency and should be treated as 1 × 10^16^ Hz [42] (UV light, considering its good performance for gas desorption [43,44]). k is the Boltzmann constant and *T* is the temperature. The required times to desorb CO and HCHO molecules from the Au-WS_2_ monolayer are calculated as 0.01 and 11.86 s at room temperature (298 K). The time is long enough to allow surface interaction and desorption under UV light, permitting another detection [42]. Conclusively, the Au-WS_2_ monolayer can be explored as a reusable and room-temperature CO and HCHO sensor to evaluate the operation status of dry-type reactors.

**Figure 5:**
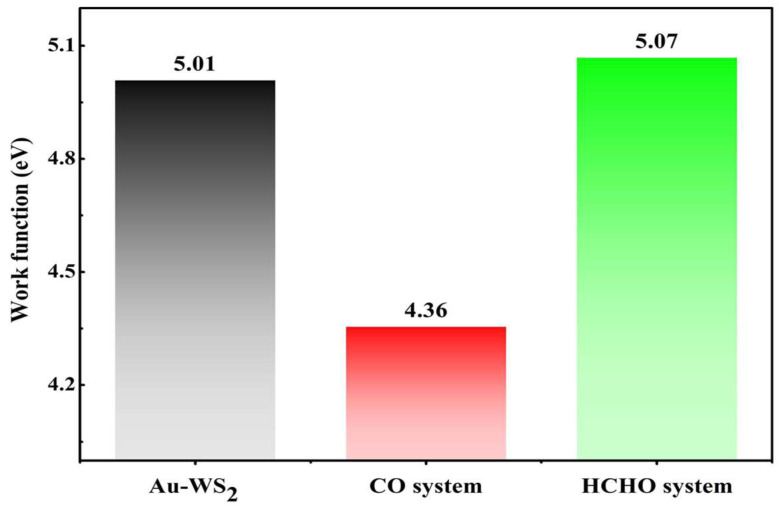
WF of freestanding Au-WS_2_ monolayer and gas adsorption systems.

For the gas–surface interaction, the work function (WF) of the nano-surface should also be explored, in order to determine whether a certain material could be used as the WF-based sensor for gas detection. Specifically, WF reflects the difficulty faced by a certain material in donating an electron out of its surface to the vacuum region. This can largely determine the charge-transfer behavior and orbital localization of the nano-surface when interacting with gas species [45]. In this report, the WFs of the freestanding Au-WS_2_ monolayer and the related gas adsorption systems are exhibited in Figure 5. From this figure, the WF of the isolated Au-WS_2_ monolayer is 5.01 eV and much larger than that of the graphene (4.60 eV [46]), which implies the stronger electron affinity of the Au-WS_2_ monolayer. In other words, the Au-WS_2_ monolayer exhibits a stronger electron-liberating property than that of the graphene. The WFs for the CO and HCHO adsorption systems are calculated to be 4.36 and 5.07 eV. Namely, the WF of Au-WS_2_ monolayer shows an obvious reduction by 13.0% after CO adsorption, and a slight increase by 1.2% after HCHO adsorption. So the Au-WS_2_ monolayer becomes more active and able to release electrons only after CO adsorption in this work. Therefore, the Au-WS_2_ monolayer could also be explored as a potential WF-based gas sensor for CO detection using the Kelvin oscillator device [47].

Via the analysis above, the use of a Au-WS_2_ monolayer as a resistance- and WF-type gas sensor is illustrated and demonstrated. It is our hope that these findings can be further verified in experimental reports. This work is also expected to stimulate the exploration of WS_2_-based materials for gas sensing and other applications, especially in the field of electrical engineering, to evaluate the operation status of certain devices such as dry-type reactors.

## 4. Conclusions

This work purposes a Au-WS_2_ monolayer as a new type of gas sensing material, and expounds its adsorption and sensing effects upon CO and HCHO molecules, in order to evaluate the operation status of dry-type reactors. The main findings are as follows:

i. The Au atom can be stably adsorbed on the T_S_ site of the pristine WS_2_ monolayer with the *E*_bind_ of −3.12 eV, making the bandgap 0.80 eV;
ii. The Au-WS_2_ monolayer performs chemisorption upon CO and HCHO molecules with *E*_ad_ values of −0.82 and −1.01 eV, respectively, in which the CO molecule behaves as an electron-donator while the HCHO molecule behaves as an electron-acceptor;
iii. The sensing responses of the Au-WS_2_ monolayer upon CO and HCHO molecules are 58.7% and −74.4%, with recovery times of 0.01 and 11.86 s, respectively, indicating its potential for use as a reusable and room-temperature gas sensor for CO and HCHO detection;
iv. The work function of the Au-WS_2_ monolayer suffers a 13.0% decrease in the CO system, while it exhibits a 1.2% increase in the HCHO system, suggesting its potential to be used as a WF-based gas sensor for CO detection.

The findings in this work are beneficial to uncovering the gas sensing potential of the Au-WS_2_ monolayer, and can shed light on further investigations into WS_2_-based gas sensors in the gas sensing field, especially for the evaluation of the operation status of dry-type reactors.

## Data Availability

The data presented in this study are available in the article.
